# Successful Treatment of Nevus of Ota With a 694-nm Q-switched Ruby Laser: A Case Report

**DOI:** 10.7759/cureus.111503

**Published:** 2026-06-25

**Authors:** Luis Velázquez Arenas, Sarahi Garay Enriquez, Daniela Gómez Guerra

**Affiliations:** 1 Dermatology, School of Medicine and Health Sciences at Tecnológico de Monterrey, Monterrey, MEX; 2 Medicine, School of Medicine and Health Sciences at Tecnológico de Monterrey, Monterrey, MEX

**Keywords:** cutaneous hyperpigmentation, dermal melanocytosis, laser-therapy, nevus of ota, qs ruby laser

## Abstract

Nevus of Ota is a benign dermal melanocytosis characterized by gray-blue facial hyperpigmentation that may cause significant cosmetic concerns and is commonly treated with laser therapy. We present the case of a 39-year-old woman with facial nevus of Ota who underwent treatment with a 694-nm Q-switched ruby laser, achieving marked pigment clearance after two treatment sessions. This case further supports the efficacy and safety of Q-switched ruby laser technology as a therapeutic option for nevus of Ota.

## Introduction

Nevus of Ota is a benign form of dermal melanocytosis characterized by a gray-blue hyperpigmentation distributed primarily along the ophthalmic and maxillary divisions of the trigeminal nerve. The condition most commonly presents unilaterally and, in addition to cutaneous involvement, pigmentation may extend to ocular and mucosal structures, including the conjunctiva, sclera, cornea, uvea, oral cavity, and nasal mucosa. Although generally asymptomatic, occasional cases with neurologic manifestations, such as sensory loss, have been described. Moreover, affected individuals have an increased risk of ocular complications, particularly glaucoma and uveal melanoma [1].

Due to the cosmetic burden associated with facial involvement, treatment is frequently sought by affected individuals. Laser therapy has become the mainstay of management, with Q-switched nanosecond lasers historically serving as the standard therapeutic approach. Advances in laser technology have led to the widespread use of pigment-selective devices, including the 755-nm alexandrite, 694-nm ruby, and 1064-nm neodymium-doped yttrium aluminum garnet lasers, all of which have demonstrated favorable efficacy profiles with significant pigment clearance and excellent cosmetic outcomes in patients with nevus of Ota [2]. Here, we present a case of nevus of Ota treated with a 694-nm Q-switched ruby laser, achieving substantial pigment reduction and excellent cosmetic improvement after two treatment sessions.

## Case presentation

A 39-year-old woman with Fitzpatrick skin phototype IV [3] presented with a congenital hyperpigmented lesion involving the left side of the face, consistent with nevus of Ota. The patient sought treatment for cosmetic improvement of the lesion and reported no previous therapeutic interventions. Physical examination revealed an irregular blue-gray to brown dermal pigmentation distributed over the left temporal region, extending toward the lateral canthus of the left eye, classified as Peking Union Medical College Hospital classification (PUMCH) type II and Tanino’s type II [4]. No scleral, conjunctival, or mucosal pigmentation was identified. Ophthalmologic evaluation revealed no evidence of glaucoma, uveal involvement, or other ocular abnormalities. Neurologic examination was unremarkable, with no sensory deficits or other associated symptoms.

**Figure 1:**
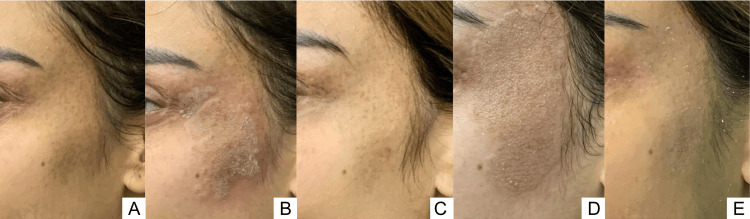
Sequential clinical evolution of nevus of Ota during treatment with a 694-nm Q-switched ruby laser (A) Baseline lesion before treatment. (B) Immediate post-treatment appearance after the first laser session. (C) One-month follow-up after the first session and before the second treatment. (D) Immediate post-treatment appearance after the second laser session. (E) One-month follow-up after completion of treatment, demonstrating substantial pigment clearance.

Treatment was performed using a Q-switched 694 nm ruby laser. During the first session, approximately 80% of the pigmented area was treated using a frequency of 1 Hz and a fluence of 4 J/cm². Immediate tissue response was characterized by the development of a gray-white frosting endpoint, indicating adequate laser energy delivery. The procedure was well tolerated, and no immediate complications were observed. At the one-month follow-up visit, a noticeable reduction in pigmentation intensity was observed, with partial clearance of the treated areas. Prior to the second treatment session, a topical anesthetic cream was applied under occlusion for one hour. Laser parameters were maintained, with the exception of a slight increase in fluence to 4.2 J/cm². The entire residual lesion was treated, again achieving appropriate frosting and immediate tissue response. One month after the second session, the patient demonstrated a significant reduction in pigmentation with favorable cosmetic improvement and no evidence of scarring, dyspigmentation, or any other adverse effects (Figure 1). The patient expressed a high degree of satisfaction with the cosmetic outcome achieved after only two treatment sessions; therefore, no additional laser treatments were performed.

## Discussion

The management of nevus of Ota has evolved considerably over recent decades, with laser therapy emerging as the treatment of choice due to its ability to achieve significant pigment clearance while maintaining a favorable safety profile. Evidence supporting this approach was notably provided by Watanabe and Takahashi, who documented the efficacy of Q-switched ruby lasers in the treatment of nevus of Ota, reporting progressive pigment clearance with increasing numbers of treatment sessions. In their series of 114 patients with nevus of Ota, excellent responses, defined as greater than 70% pigment lightening, were achieved in 33 of 35 patients who underwent four to five treatment sessions. In contrast, only 4 of 31 patients treated with three sessions and 2 of 25 patients treated with two sessions achieved comparable clearance [5]. Notably, our patient demonstrated substantial pigment reduction after only two treatment sessions, highlighting the potential of Q-switched ruby lasers to achieve favorable outcomes.

Jiang et al. further demonstrated the efficacy of Q-switched ruby lasers in pediatric patients with nevus of Ota, reporting significant clinical improvement in more than 90% of treated individuals. Importantly, younger children achieved favorable outcomes with fewer treatment sessions and a lower incidence of adverse events compared with older children, supporting the benefits of early intervention [6].

On the other hand, Zhang et al., in a systematic review of laser-based therapies for benign pigmented lesions, analyzed 29 studies evaluating nevus of Ota, acquired bilateral nevus of Ota-like macules, and Hori’s nevus. Nd: YAG lasers were the most commonly studied modality, accounting for 12 studies, followed by Q-switched ruby lasers with 9 studies and picosecond alexandrite lasers with 7 studies. Among the studies evaluating Q-switched ruby lasers, successful outcomes were reported in 88.9%, supporting their longstanding role in the management of dermal melanocytosis. Reported adverse effects were predominantly mild and transient, with post-inflammatory hyperpigmentation and erythema being the most frequently observed complications [7].

## Conclusions

The present case demonstrates that treatment of nevus of Ota with the 694-nm Q-switched ruby laser can achieve marked pigment clearance and excellent cosmetic outcomes after only two treatment sessions. This response is particularly noteworthy, as nevus of Ota typically requires multiple laser sessions to attain comparable levels of clinical improvement. The substantial reduction in pigmentation observed in this patient after a limited number of treatments highlights the efficacy of this modality in selected cases. These findings are consistent with the growing body of evidence supporting laser therapy as the standard of care for dermal melanocytosis and further reinforce the role of the 694-nm Q-switched ruby laser as an effective therapeutic option. Nevertheless, larger studies with extended follow-up are warranted to better characterize the factors associated with rapid treatment response and to further establish the long-term efficacy and safety profile of this approach.
